# Effect of Inherent Mg/Ti Interface Structure on Element Segregation and Bonding Behavior: An Ab Initio Study

**DOI:** 10.3390/ma18020409

**Published:** 2025-01-16

**Authors:** Xiaodong Zhu, Kaiming Cheng, Jin Wang, Jianbo Li, Jingya Wang, Huan Yu, Jixue Zhou, Yong Du

**Affiliations:** 1 Shandong Provincial Key Laboratory of High Strength Lightweight Metallic Materials, Advanced Materials Institute, Qilu University of Technology (Shandong Academy of Sciences), Jinan 250014, China; 10431220138@stu.qlu.edu.cn (X.Z.); yuhuan@sdas.org (H.Y.); 2 School of Materials Science and Engineering, Chongqing University, Chongqing 400044, China; lijianbo1205@163.com; 3 National Engineering Research Center of Light Alloy Net Forming and State Key Laboratory of Metal Matrix Composite, Shanghai Jiao Tong University, Shanghai 200240, China; jingya.wang@sjtu.edu.cn; 4 State Key Laboratory of Powder Metallurgy, Central South University, Changsha 410083, China; yong-du@csu.edu.cn

**Keywords:** inherent Mg/Ti interface, ab initio molecular dynamics (AIMD), rare-earth elements (Gd, Y), elemental segregation, Griffith work of fracture

## Abstract

To provide insight into the interface structure in Ti particle-reinforced Mg matrix composites, this study investigates the inherent Mg/Ti interface structure formed during the solidification of supercooled Mg melt on a (0001)Ti substrate using ab initio molecular dynamics (AIMD) simulations and density function theory (DFT) calculation. The resulting interface exhibits an orientation relationship of ${\left(0001\right)}_{\mathrm{Mg}}{//\left(0001\right)}_{\mathrm{Ti}}$ with a lattice mismatch of approximately 8%. Detailed characterizations reveal the occurrences of ${\left(0001\right)}_{\mathrm{Mg}}$ plane rotation and vacancy formation to overcome the lattice mismatch at the inherent Mg/Ti interface while allowing Mg atoms to occupy the energetically favorable hollow sites above the Ti atomic layer. The atomic diffusion behaviors of rare-earth elements Gd and Y at the Mg/Ti interface was examined using the climbing image nudged elastic band (CI-NEB) method, demonstrating a strong segregation tendency towards the interface promoted by the inherent interface structure. Additionally, the calculated Griffith work indicates enhanced interfacial adhesion due to the segregation of Gd and Y, which is beneficial for the mechanical properties of the composite.

## 1. Introduction

Magnesium-based materials have attracted tremendous attention in various industrial applications due to their excellent structural and functional properties. Their low density, combined with high specific strength and high energy density, makes them highly attractive for using in sectors such as transportation [1], aerospace [2], electronics [3], and energy [4]. However, the application of magnesium (Mg) alloys is hindered by the inherent limitations, including low strength, poor elastic modulus, limited ductility [5], and the issue of passivation layer formation on the Mg anode [6]. The mechanical properties of Mg alloys can be effectively improved by alloying, where the most commonly used elements include Al, Zn, Zr, Y, and Gd [7]. The purification of magnesium and its alloys should be treated carefully due to the considerable influence on the mechanical properties and corrosion behavior [8]. Meanwhile, different processes, such as casting [9,10,11,12], plastic deformation [13], and welding [14], are often introduced due to various applications of magnesium alloys. In addition, the incorporation of reinforcing particles, forming magnesium matrix composites (MgMCs), represents another important strategy to enhance the mechanical properties of magnesium alloys [15]. Typically, particles of carbides [16,17,18,19], oxides [20,21], borides [22,23,24], and metallic particles [25,26,27,28] can be used as reinforcements of MgMCs. Among these, titanium (Ti) particles present as a promising candidate for the reinforcement of magnesium alloys, considering their beneficial characteristics such as low density, high specific strength, exceptional ductility, and a hexagonal close-packed (HCP) structure with the potential to form coherent interface with Mg [29].

The mechanical properties of Ti particle-reinforced Mg composites are significantly affected by the interfacial structures [30]. The Mg/Ti interfacial structure should be understood from two levels, i.e., the orientation relation and the local atom arrangement following a specific orientation relation. So far, various orientation relations of the Mg/Ti interface have been experimentally observed. G. Garcés et al. [31,32] detected specific orientations between the precipitated Ti particles and the Mg matrix, i.e., ${\left[0001\right]}_{\mathrm{Mg}}//{\left[0001\right]}_{\mathrm{Ti}}$, and ${\left[11\overset{-}{2}0\right]}_{\mathrm{Mg}}//{\left[11\overset{-}{2}0\right]}_{\mathrm{Ti}}$, based on the annealing of the Mg-12 wt.% Ti alloy fabricated by the physical vapor deposition (PVD) method. These precipitates tend to lose their coherence as the annealing time increases. Similar possible matching planes, i.e., ${\left\{0002\right\}}_{\mathrm{Mg}}//{\left\{0002\right\}}_{\mathrm{Ti}}$, were predicted by Xiao et al. [33] between Ti particles and the Mg matrix using the theoretical edge-to-edge matching model [34,35]. However, this interfacial structure was not confirmed in their experimental investigation, while the orientation matching relationship of ${\left(01\overset{-}{1}0\right)}_{\mathrm{Mg}}//{\left(1\overset{-}{2}1\overset{-}{3}\right)}_{\mathrm{Ti}}$ and $\;{\left(0002\right)}_{\mathrm{Mg}}//{\left(10\overset{-}{1}0\right)}_{\mathrm{Ti}}$ were found in some grains of Ti polycrystal particles. Yu et al. [36,37,38] adopted powder metallurgy to fabricate Ti/AZ31 and Ti/AZ61 composites, which exhibited a coherent interface on the (101) and (100) crystal planes and a semi-coherent interface on the (002) crystal plane. Pu et al. [39,40] prepared Ti/VW94 composites by semi-solid-state stirring-assisted ultrasonic vibration and hot extrusion, and likewise revealed several coherent boundaries of ${\left(10\overset{-}{1}0\right)}_{\mathrm{Mg}}//{\left(0002\right)}_{\mathrm{Ti}}$ and ${\left(0002\right)}_{\mathrm{Mg}}//{\left(10\overset{-}{1}0\right)}_{\mathrm{Ti}}$.

However, investigation on the Mg/Ti interface at the level of local atom arrangement is often experimentally difficult. The first-principles calculations based on density functional theory (DFT) play an important role in providing a feasible route [16,23,24]. Wu et al. [41] focused on the predefined orientation relationships of ${\left(0001\right)}_{\mathrm{Mg}}{//\left(0001\right)}_{\mathrm{Ti}}$, and proposed a Mg/Ti interface model using the “stress balancing” approach. This approach aims at minimizing the lattice mismatch, where an optimal interface match of 7:8 was suggested for the twist Mg/Ti interface based on the calculation of interfacial energy and work of adhesion. Bao et al. [42] also chose the orientation relationship of ${\left(0001\right)}_{\mathrm{Mg}}{//\left(0001\right)}_{\mathrm{Ti}}$ and optimized the interfacial configuration by evaluating the work of adhesion and interface energy. The results revealed that the interface, where Mg atoms occupy the hollow site of the topmost Ti atom layer, was energetically favorable. Notably, the contradiction between the two models lies in the fact that the twist Mg/Ti interface to accommodate lattice mismatch (Wu’s model) should lead to Mg atoms unavoidably being located on the top site of Ti atoms, which is energetically unfavorable according to Bao’s model. So far, the inherent atomic structure at the Mg/Ti interface remains unclear even for the low-index orientation relationships of ${\left(0001\right)}_{\mathrm{Mg}}{//\left(0001\right)}_{\mathrm{Ti}}$.

In addition, Mg/Ti interfaces within Mg-based metal composites (MgMCs) are susceptible to the influence of alloying elements in the Mg matrix, as these interfaces are often considered as high-energy regions prone to elemental diffusion, chemical reaction, and the segregation of alloying elements and impurities [30,43]. So far, a number of computational modeling and experimental observations have been performed for various types of Ti particle-reinforced MgMCs, representing a good combination for the understanding the element distribution behavior within the interface region. For example, intermetallic compounds of the Ti-Al system were both experimentally detected [36,37,38] and computationally predicted [44] to generate at the interface due to the reaction between Ti particles and Al dissolved in the AZ matrix. Rare-earth (RE) elements, as a serial of important alloying elements, were experimentally observed to segregate on the Mg side of the Mg/Ti interface [15,39,40], which has also been computationally investigated [45]. And the segregation of RE elements dissolved in the Mg matrix is often considered as a contributor to the enhancement of interface bonding. In addition, by using first-principles calculations, numerous studies have been conducted to investigate the interfacial properties between the Mg matrix and other types of reinforcing particles, such as Mg/SiC [16], Mg/WC [46], and Mg/MgZn_2_ [47]. Although the atom diffusion behavior of alloying elements from the matrix to the interface is essential for understanding the tendency of element segregation, the effect of the inherent Mg/Ti interface structure on promoting atom diffusion is still not clarified.

Therefore, the current work attempts to determine the inherent Mg/Ti interfacial structure formed in the solidification process of supercooled Mg on the (0001)_Ti_ plane and clarify its effect on promoting the atom diffusion of alloying elements with the aid of the ab initio molecular dynamics (AIMD) simulations and density functional theory (DFT) calculations. The inherent interfacial structure is analyzed on the atomistic scale, and the occupancy mechanism of Mg atoms at the Mg/Ti interface is uncovered. Following the occupancy mechanism, the atom diffusion of RE elements (Gd and Y) at the Mg/Ti interface is investigated by the climbing image nudged elastic band (CI-NEB) method, and the RE-affected structural stability of the Mg/Ti interface is discussed according to the Griffith work of fracture. It is expected that the current study can provide a valuable insight into the atomic-scale solidification and structural behavior in Mg/Ti systems, contributing to the development of advanced Mg-based composites with enhanced performance.

## 2. Materials and Methods

The AIMD simulations and DFT calculations were carried out using the Vienna Ab initio Simulation Package (VASP 5.4.4) [48,49]. The visualization of the structural models was conducted using VESTA (Ver. 3.4.3) [50] and OVITO (Ver. 3.8.4) [51] software. The interaction between ion cores and valence electrons was determined by projector augmented wave (PAW) potentials [52,53], and the electronic exchange–correlation was described by the generalized gradient approximation (GGA) with Perdew–Burke–Ernzerhof (PBE) functionals [54]. AIMD was used to simulate the solidification process. The Brillouin zone was sampled using only the Gamma (Γ) point in AIMD simulations. NVT ensemble with Nosé–Hoover thermostat [55,56] was applied, and the timestep was set as 2 fs. In the present work, all the calculations of the interfacial properties were based on the framework of density functional theory. For the DFT calculation, the plane-wave cutoff energy was set to 500 eV, and the Brillouin zone was sampled using the K-point mesh of 3 × 5 × 1 in the Gamma scheme. The total energy was converged to within 10^−6^ eV per atom, and the force on each atom was converged to within 0.02 eV/Å.

The saddle point and the corresponding minimum energy pathway (MEP) were determined using the CI-NEB method [57] with VASP serving as the computational engine. The CI-NEB calculations were conducted by following the settings of the DFT calculation as mentioned above, except for the energy convergence criterion that was reset to 0.03 eV. No fewer than three images, between the initial and final positions, were selected to identify the saddle points along the MEP.

## 3. Results and Discussion

### 3.1. Inherent Mg/Ti Interface Structure Formed During Solidification Process of Supercooled Liquid Mg on (0001)_Ti_ Plane

To investigate the inherent Mg/Ti interface structure formed during the solidification process of supercooled liquid Mg on the (0001) plane of Ti, a 4 × 4 × 3 supercell of HCP Ti was constructed as the Ti substrate with six atom layers, and a supercooled Mg droplet containing 96 atoms was kept 2 Å away from the (0001)_Ti_ plane. The supercooled Mg droplet configuration was generated using a Monte Carlo algorithm, relaxed at 973 K for 5 ps, and then cooled to 900 K at a rate of 1 K/fs, with a final relaxation of 1 ps. To prevent the interactions between periodic images perpendicular to the interface, a vacuum region more than 10 Å was attached to the Mg droplet. The AIMD simulation protocol involved maintaining the initial temperature at 900 K for 20 ps. This was followed by a step-wise cooling process, reducing the temperature to 700 K, 500 K, and finally 300 K, with each temperature held for 10 ps.

**Figure 1:**
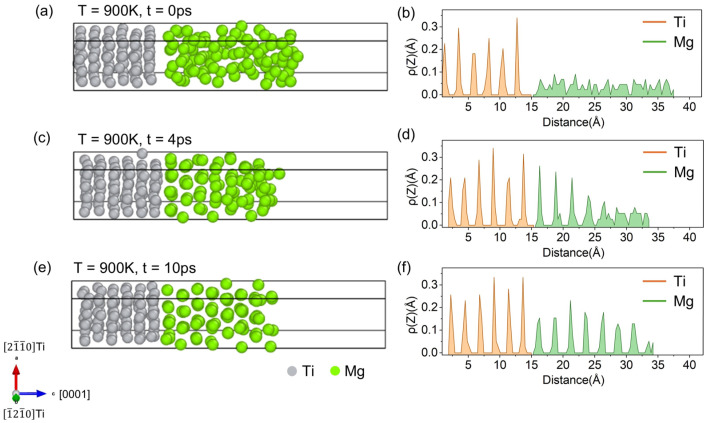
Configuration and *Z*-axis density profiles ρ(z) of the Mg melt solidified on the (0001)_Ti_ plane at 900 K. (**a**) Initial state configuration of the interface model; (**b**) corresponding *Z*-axis density profile (ρ(z)) at the initial state. (**c**) Configuration of the interface model after 4 ps; (**d**) *Z*-axis density profile (ρ(z)) after 4 ps. (**e**) Configuration of the interface model after 10 ps; (**f**) *Z*-axis density profile (ρ(z)) after 10 ps.

The atomic density profile, ρ(z), along the *c*-axis ([0001]_Ti_ direction) was analyzed using the slices parallel to the interface, as described in previous studies [58,59]. The ρ(z) function was described using the following formula:

(1) $$\varrho (z)=\frac{\left\langle N_{Z}\right\rangle }{S\Delta z}$$

where S represents the area of the interface. Δz is the width of each slice, and denotes the atomic count averaged over time within the range from z − Δz/2 to z + Δz/2. The initial relaxation configuration and the density profile ρ(z) at 900 K are illustrated in Figure 1b. The six Ti layers display a regular pattern of oscillating peaks, demonstrating a typical ordered HCP structure. In contrast, the c-axis density distribution of Mg atoms shows irregular fluctuations, indicating a liquid-phase state. The fluctuations correspond to the random structure of the Mg melt. As shown in Figure 1d, a three-layered ordered structure of Mg is formed near the Mg-Ti interface after 4 ps at 900 K. Several weak peaks also appear at the solidification front, indicating the formation of solid-like structures within the remaining liquid Mg. After an additional 6 ps, as depicted in Figure 1f, the Mg region shows a fully ordered structure, which remains stable for a further 10 ps.

**Figure 2:**
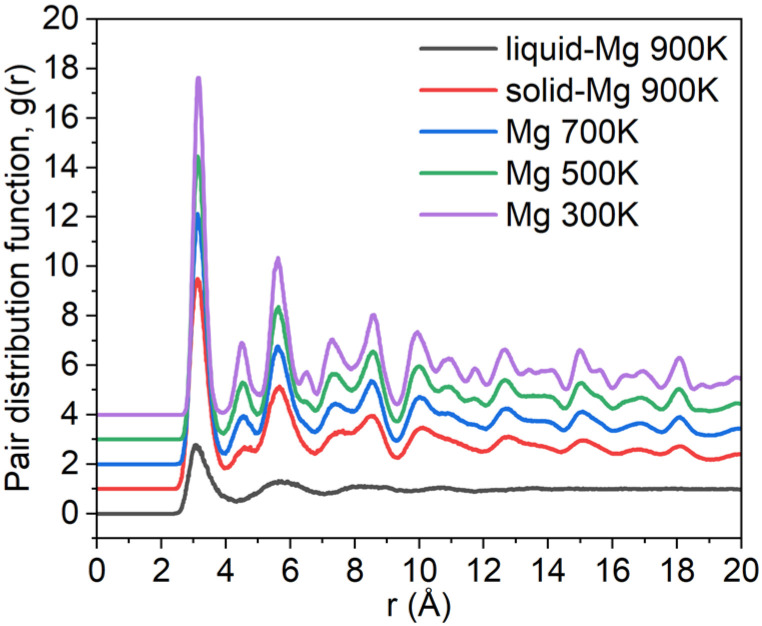
Pair distribution function (PDF) of Mg atoms on Ti substrate.

In Figure 2, the structural ordering of the Mg region at the interface from 900 K to 300 K is further examined by the pair distribution function (PDF). PDF is obtained using the following equation [60]:

(2) $$g_{A–B}(r)=\frac{V}{N_{A}N_{B}}\langle \sum_{i=1}^{N_{A}}\frac{n_{\mathrm{iB}}\left(r,\Delta r\right)}{4\pi r^{2}\Delta r}\rangle$$

where V is the supercell volume, N_A_ and N_B_ are the atom number of A and B, respectively, and n_iB_ is the atom number of B in the sphere shell from r to r + Δr around the ith A atom. As the temperature decreases, three main features are observed: (i) the intensification of the first peaks, indicating short-range ordering within the Mg region; (ii) the emergence of new peaks in the first and second valleys, suggesting medium-range ordering; and (iii) the splitting of the third peak, indicative of long-range ordering in the solidified Mg structure.

**Figure 3:**
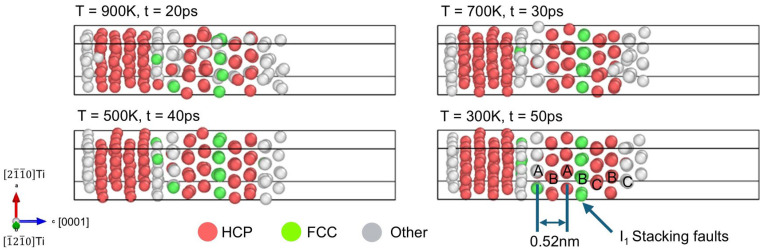
Common neighbor analysis of Mg melts during solidification.

To further characterize the ordered structure obtained from the AIMD simulations, common neighbor analysis [61] was performed at 900 K, 700 K, 500 K, and 300 K, as shown in Figure 3. The results indicate that as the temperature decreases, an increasing number of Mg atoms are identified as HCP structures, corresponding to the stable phase of Mg. Additionally, Figure 3 reveals the parallel orientation between the (0001) plane of Mg and Ti, which is further evidenced by the interplanar spacings of the Mg solidified structure at 300 K, measured to be 0.52 nm, consistent with the lattice parameter c of Mg. Interestingly, a rotation of the ${\left(0001\right)}_{\mathrm{Mg}}$ plane around the c-axis and the presence of I_1_-type stacking fault with an ABABCBC sequence are observed upon solidification on the ${\left(0001\right)}_{\mathrm{Ti}}$ plane [62]. This can be attributed to lattice mismatch and stress relaxation, as described by the epitaxial nucleation model [63]. Since Mg has a lower elastic modulus than Ti, most of the strain to accommodate the lattice mismatch occurs within the solidified Mg. Future simulations on an enlarged Ti substrate will provide more insights into the detailed structure of the inherent Mg/Ti interface in the next section.

From a nucleation point of view, as shown in Figure 1, the formation of an inherent Mg/Ti interface structure during the solidification of the supercooled Mg droplet on the Ti substrate reveals a layer-by-layer ordering process at 900 K, starting from the Mg-Ti interface. The ${\left(0001\right)}_{\mathrm{Ti}}$ plane offers low-energy sites for adjacent Mg liquid atoms to form the initial ${\left(0001\right)}_{\mathrm{Mg}}$ layer, which promotes the subsequent formation of an HCP structure. Previous experiments [31,32] and theoretical model predictions [33] have reported the equivalent orientation relationship, such as ${\left[0001\right]}_{\mathrm{Mg}}//{\left[0001\right]}_{\mathrm{Ti}}$ and ${\left\{0002\right\}}_{\mathrm{Mg}}//{\left\{0002\right\}}_{\mathrm{Ti}}$, with an interfacial lattice mismatch of 9% and 10%, respectively, indicating the facilitation of heterogeneous nucleation by the substrate. In this study, a similar lattice mismatch of about 8% is measured at the Mg/Ti interface, illustrating that the Ti particle has the potential to be the effective grain refiner for MgMCs [29].

### 3.2. Role of Vacancy Formation on Accommodating Lattice Mismatch at Inherent Mg/Ti Interface

The current AIMD simulation model consisted of a Ti substrate composed of six layers with an 8 × 9 HCP structure, a 96-atom magnesium droplet generated by the same way as in Section 3.1, and a vacuum layer exceeding 10 Å attached to the Mg droplet. The system was then cooled from 900 K to 700 K and subsequently to 300 K, with each temperature maintained for 10 ps to observe the solidification process.

**Figure 4:**
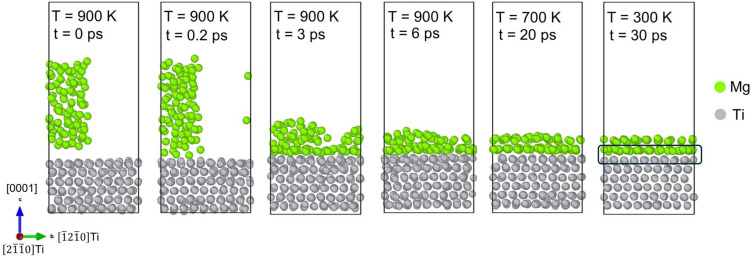
Side-view snapshots capturing the solidification of the Mg melt on the (0001)_Ti_ plane at various temperatures and times.

Figure 4 shows a series of side-view snapshots captured in the formation of an inherent Mg/Ti interface structure during the solidification process of the Mg droplet on the (0001)_Ti_ plane at various temperatures and times. At 900 K, the Mg droplet first makes contact with the Ti surface at approximately 0.2 ps. The first ordered Mg layer on the Ti surface is formed by 3 ps, and a complete spreading of Mg atoms across the Ti surface is achieved by 6 ps. Upon cooling to 700 K, there is a noticeable improvement in the ordering of the second Mg layer at 20 ps. When the temperature reaches 300 K, the fully spreading and stacking of Mg atoms in the second layer occur, indicating a fully developed epitaxial interface.

**Figure 5:**
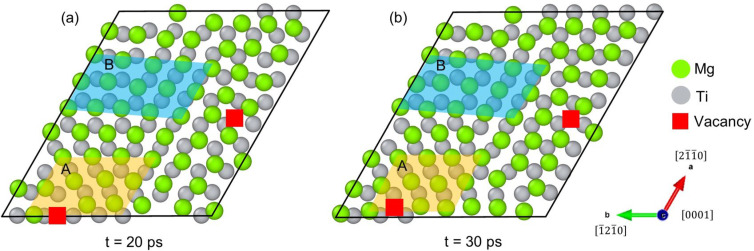
Cross-sections of the Mg-Ti interfacial structure at (**a**) 20 ps and (**b**) 30 ps: (Region A) interface model vacancy at a particular location; (Region B) interface model with the rotation of the (0001)_Mg_ planes.

Figure 5 presents a detailed view of the inherent Mg/Ti interface at 300 K within a 10 ps simulation interval. The rotation of the (0001)_Mg_ plane is evident in this cross-section view, and Mg atoms preferentially occupy the positions above the hollow sites among Ti atoms. To overcome the interfacial lattice mismatch, Wu et al. [41] previously constructed a Mg/Ti interface model by rotating the (0001)_Mg_ plane relative to the (0001)_Ti_ plane. In our AIMD simulations, the rotation of (0001)_Mg_ plane relative to the (0001)_Ti_ plane occurs naturally during the solidification of supercooled liquid Mg on the (0001)_Ti_ plane, which demonstrates that rotation is an effective behavior to overcome the lattice mismatch and stabilize the interface.

Regions A and B in Figure 5 illustrate two local interfacial areas, one with vacancies (Region A) and one without (Region B). The rotation of the Mg arrangement in Region A is significantly less pronounced than in Region B, suggesting that the presence of vacancies can also help accommodate lattice mismatch. It can be further pointed out from Figure 5 that both vacancy formation and lattice rotation remain stable within the simulation time. To evaluate the role of vacancies in reducing lattice mismatch, two simplified local Mg/Ti interface models were constructed, namely, Wu’s rotation model and the current vacancy model, as shown in Figure S1, and the Griffith work of adhesion for each model was calculated using the following equation [64]:

(3) $$W_{G}=\left[\begin{array}{c} E_{1\text{-}\mathrm{surface}}+E_{2\text{-}\mathrm{surface}}\;- \end{array}E_{1\text{-}2\text{-}\mathrm{interface}}\right]/A$$

where $E_{1\text{-}2\text{-}\mathrm{interface}}$ denotes the total energy of the interface model, $E_{1\text{-}\mathrm{surface}}$ and $E_{2\text{-}\mathrm{surface}}$ represent the total energy of the isolated Ti and Mg slabs, respectively, and A is the area of the interface or the corresponding free surfaces. The Griffith work provides a measure of the chemical bonding strength at the interface. The vacancy model has higher Griffith work (2.15 J/m^2^) than Wu’s rotation model (2.06 J/m^2^). To further elaborate the thermodynamic stability of the interface, the interface energy (*γ*) of all considered interface models was evaluated by the following formula [47]:

$$\gamma_{\mathrm{int}}=(1/A)\left[\begin{array}{c} E_{\mathrm{Mg}/\mathrm{Ti}}-N_{\mathrm{Mg}}\mu_{\mathrm{Mg}}-N_{\mathrm{Ti}}\mu_{\mathrm{Ti}} \end{array}\right]-\sigma_{\mathrm{Mg}}-\sigma_{\mathrm{Ti}}$$

where $E_{\mathrm{Mg}/\mathrm{Ti}}$ is the total energy of the interface model. $\mu_{\mathrm{Mg}}$ and $\mu_{\mathrm{Ti}}$ are the chemical potentials of Mg and Ti atoms in bulk, respectively. $N_{\mathrm{Mg}}$ and $N_{\mathrm{Ti}}$ represent the numbers of Mg and Ti atoms in the interface model, respectively. $\sigma_{\mathrm{Mg}}$ and $\sigma_{\mathrm{Ti}}$ represent the surface energy of the (0001)_Mg_ plane and the (0001)_Ti_ plane, respectively. Based on our calculations, the interfacial energy of the vacancy model is 1.10 J/m^2^, and the interfacial energy of Wu’s rotation model is 0.72 J/m^2^.

Now, we can discuss on the interrelation between current rotation- and vacancy-mediated models. Based on the calculated Griffith work and interface energy, the interface bonding strength of the vacancy model is higher than that of the rotation model, but the interface stability is the opposite. If the accommodation of lattice mismatch between Mg and Ti only relies on lattice rotation, it is inevitable that there are Mg atoms located on the top site of the topmost Ti atom layer, which is unfavorable in energetics according to Bao et al.’s work [42]. To avoid such an energetically unfavorable phenomenon, the local arrangement of Mg atoms should require the formation of vacancies to ensure their occupying the hollow site of the topmost Ti atom layer within the inherent interface. This hypothesis is consistent with current calculation result that the interface energy of the rotation model is smaller, implying that vacancy formation is a consequence of lattice rotation. Furthermore, the current vacancy formation model resolves previous contradictions between Wu’s (rotation) and Bao’s (without rotation) model. In terms of the interfacial energy, the Mg/Ti interface presents a lower value of 0.5 J/m^2^ with rotation [41] versus 0.573 J/m^2^ without rotation [42], implying the higher stability of the rotation-mediated lattice model. This can be explained according the current result that rotation is an effective behavior to overcome the lattice mismatch and stabilize the interface. On the other hand, Bao’s Mg/Ti interface model can be regarded as in a metastable state, which neglects the lattice rotation and the resulting vacancy formation. Overall, the current calculation result indicates that both lattice rotation and vacancy formation are two highly interrelated factors to overcome lattice mismatch and stabilize the Mg/Ti interface. These characterize the local atom arrangement following the specific orientation relation of ${\left(0001\right)}_{\mathrm{Mg}}{//\left(0001\right)}_{\mathrm{Ti}}$ at the inherent Mg/Ti interface.

### 3.3. Vacancy-Mediated Element Segregation and Bonding Strength of Mg/Ti Interface

As shown in Region B of Figure 5, the local atomic structure at the inherent Mg/Ti interface with vacancy formation hardly exhibits lattice rotation, which allows us to investigate vacancy-mediated element diffusion within the interfacial region. To mimic such a locally equilibrium atomic configuration, a simplified vacancy model of the Mg/Ti interface was constructed for the DFT calculation of the migration energy barriers of doping atoms, specifically Mg, Gd, and Y, using the CI-NEB method. Each model contains five layers of (0001)_Mg_ slabs and five layers of (0001)_Ti_ slabs, stacked periodically along the c-axis with a 10 Å vacuum layer. Interface Mg atoms are located above the hollow sites among Ti atoms and the optimal number of Mg and Ti layers was determined by calculating the surface energy of convergence with increasing slab thickness. As shown in Tables S2 and S3, the surface energy converges to 0.559 J/m^2^ with N = 5 for the (0001)_Mg_ slab, which agrees with other GGA results (0.56 J/m^2^) [65]. The surface energy of the (0001)_Ti_ surface with five layers in the present work is converged to 1.96 J/m^2^. Our present data are in accordance with the previous calculated value of 1.94 J/m^2^ [66] and the experimental value of 1.99 J/m^2^ [67]. Therefore, the five-layer slab setup provides a bulk-like interior for the following calculations. The initial vacancy is located on the Mg bulk side of the interface.

**Figure 6:**
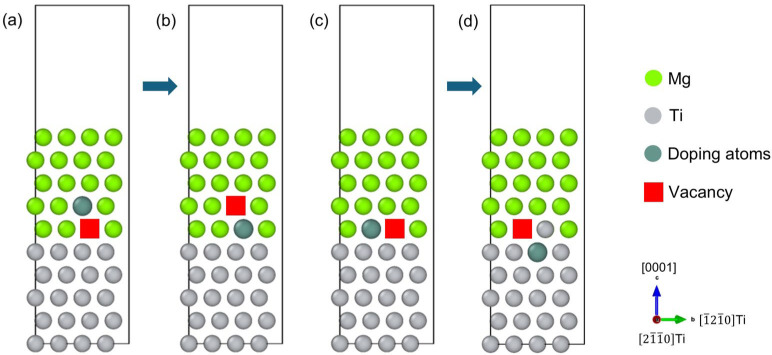
Schematic diagram of the vacancy-mediated migration behavior for the doping atoms at the Mg/Ti interface: (**a**,**b**) are the initial and final states of diffusion from the Mg matrix to the Mg side of the Mg/Ti interface, and (**c**,**d**) are the initial and final states of diffusion from the Mg side to the Ti side of the Mg/Ti interface.

**Figure 7:**
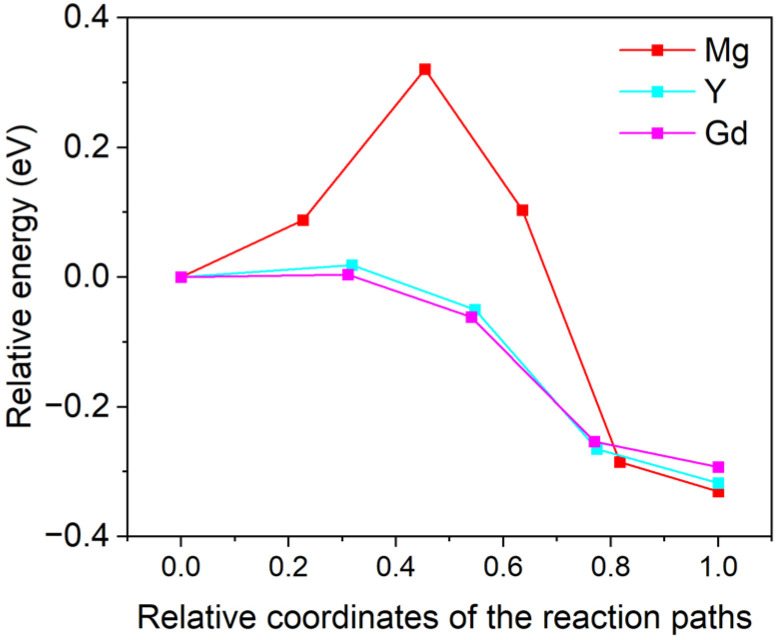
Migration energy barriers for doping elements (Mg, Gd, and Y) moving from the Mg matrix to the Mg/Ti interface.

**Figure 8:**
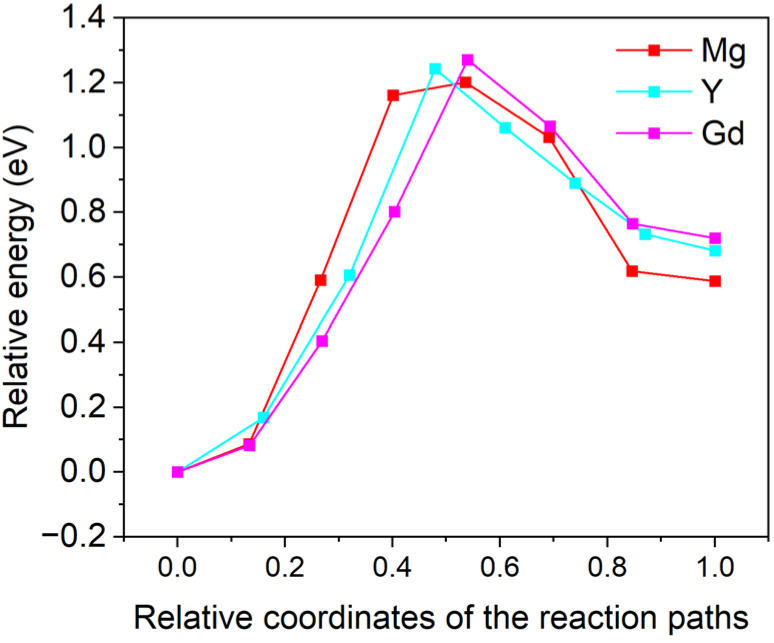
Migration energy barriers for doping elements (Mg, Gd, and Y) moving from the Mg side of the Mg/Ti interface to the Ti matrix.

Figure 6 gives a schematic diagram of the migration behaviors of the doping atoms at the Mg/Ti interface: the green spheres represent Mg atoms, the gray spheres represent Ti atoms, and the cyan spheres represent the doping atoms (Mg, Gd, and Y) diffusing within the interfacial region. Figure 6a,b illustrate the initial and final states of the doping atoms diffusing from the Mg matrix to the Mg side of the Mg/Ti interface, while Figure 6c,d depict the diffusion of the doping atoms from the Mg side of the Mg/Ti interface to the Ti side. Figure 7 shows the computed migration energy barriers of the doping atoms moving from the Mg matrix towards the Mg side of the Mg/Ti interface. The decreases in total energies from the initial states to the final ones suggest that the final states are more stable than the initial ones. The migration energy barriers of Gd (0.003 eV) and Y (0.018 eV) are significantly lower than that of Mg (0.320 eV), indicating that the migration of RE atoms (Gd and Y) to the interfacial defect can be activated more easily than that of Mg. Figure 8 compares the migration energy barriers of the doping atoms moving from the Mg side of the Mg/Ti interface to the Ti side. The migration energy barriers of Mg, Gd, and Y are 1.206 eV, 1.270 eV, and 1.242 eV, respectively, which are significantly larger than those of atoms migrating from the Mg matrix towards the Mg side of the Mg/Ti interface. Moreover, the increment in total energies from the initial states to the final ones further confirms that the vacancy-mediated element diffusion from the Mg side of Mg/Ti interface to the Ti-side is an unfavorable process. Therefore, it can be concluded that the doping RE atoms are more likely to accumulate on the Mg side of the Mg/Ti interface rather than migrating to the Ti side. This finding aligns with the experimental observation of RE segregation near Ti particles in Mg-Ti composites [40,41].

**Figure 9:**
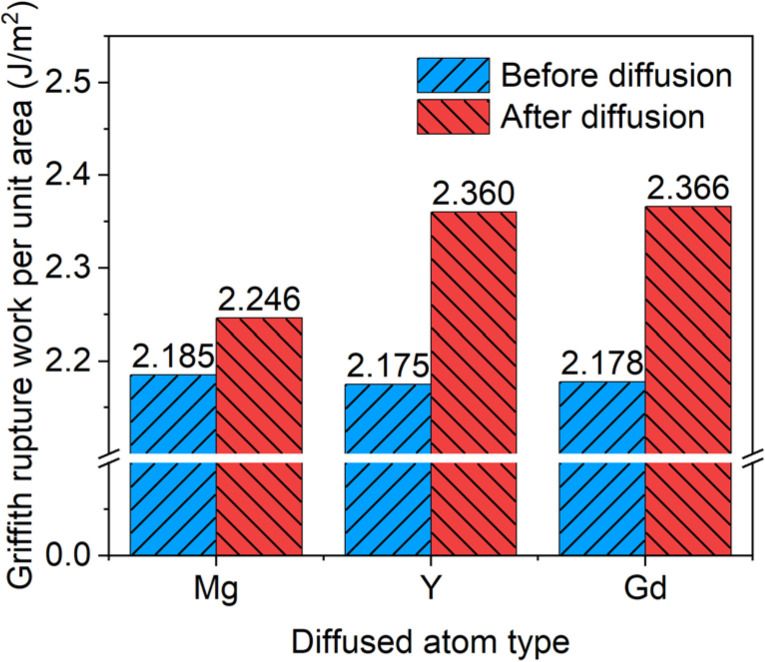
Griffith work of fracture at Mg/Ti interface before and after diffusion for different types of diffusing atoms.

The Griffith work changes caused by the migration of the doping atoms (Mg, Gd, and Y) from the Mg matrix towards the Mg side of the Mg/Ti interface are presented in Figure 9. The “before diffusion” column corresponds to the initial state as depicted in Figure 6a, and the “after diffusion” column corresponds to the final state shown in Figure 6b. The Mg/Ti interface demonstrates stronger chemical bonding after the diffusions of the doping atoms, as indicated by the higher Griffith works. Specifically, before diffusion, the addition of rare-earth atoms has little effect on the Griffith work for the Mg/Ti interface. Compared to the diffusion of Mg, the diffusion of Gd and Y causes a more significant increase in Griffith work, by about 9%, while Mg only increases by 3%. In other heterogeneous interfaces, such as Mg/MgZn_2_ [47], the doping of Y elements at the interface results in an approximately 10% increase in Griffith work, which is similar to our findings. Overall, for the Mg/Ti interface, the increase in Griffith work due to the diffusion of Gd or Y is greater than that due to the diffusion of Mg. This suggests that the segregation of RE elements, such as Gd and Y, to the Mg/Ti interface has a more pronounced positive effect on the interfacial adhesion.

## 4. Conclusions

In summary, by performing an atomic-scale simulation of the Mg/Ti interface using first-principles calculations, the inherent interfacial structure after solidification and the stability of the Mg/Ti heterogeneous interface are investigated. The segregation of rare-earth elements and their effect on interface bonding at the interface are explored based on transition state theory. The conclusions are as follows:

- With the aid of AIMD simulations, the solidification process of a supercooled Mg droplet on the (0001)_Ti_ plane was systematically investigated. At 900 K, the supercooled Mg droplet solidified in a layer-by-layer manner, forming the HCP structure and I1-type stacking faults. The interfacial orientation relationship, i.e., ${\left(0001\right)}_{\mathrm{Mg}}//{\left(0001\right)}_{\mathrm{Ti}}$, was naturally established during the solidification and remained stable as the temperature decreased from 900 K to 300 K.
- To overcome the lattice mismatch at the solidified inherent Mg/Ti interface, two main structural modifications behaviors were observed, namely, the rotation of the (0001)_Mg_ plane around the c-axis and vacancy formation. Both characters should obey the mechanism that Mg atoms preferentially occupy the positions above the hollow sites in the Ti atomic layer.
- Based on the Mg/Ti interface structure constructed following the local atomic arrangement and vacancy formation, the effect of inherent interface structure on the diffusion behaviors of Mg, Gd, and Y were examined by the CI-NEB method. The results indicated that RE elements prefer to accumulate on the Mg side of the Mg/Ti interface rather than migrate into the Ti side. The calculation of Griffith work further demonstrated that the segregation of RE elements on the Mg side of the Mg/Ti interface enhances interfacial adhesion within MgMCs.

It is worth further pointing out that the current work reveals a route of constructing an inherent solid–solid Mg/Ti interface structure by solidifying a Mg melt on the Ti substrate. The advantage of this process is to avoid the problems of optimizing the possible interfacial stacking sequences and equilibrium interfacial distance during the artificial construction of an atomic interface model. However, the current work only focuses on the inherent interface structure between Mg and the (0001)_Ti_ plane. It is expected the method can be extended to more orientations of Ti substrates ($(11\bar{2}0),\;(10\bar{1}0)$, etc.) to provide valuable insights for the development of Mg alloy particle-reinforced composite materials.

## Data Availability

The original contributions presented in this study are included in the article/Supplementary Materials. Further inquiries can be directed to the corresponding authors.

## Supplementary Materials

The following supporting information can be downloaded at: https://www.mdpi.com/article/10.3390/ma18020409/s1, Figure S1: Cross section of Mg-Ti interface: (a) rotation model, (b) vacancy model; Table S1: Calculated Griffith work for rotation model and vacancy model; Table S2: Calculated interface energy for rotation model and vacancy model; Table S3: Calculated surface energies for Mg (0001) with respect to the number of layers; Table S4: Calculated surface energies for Ti (0001) with respect to the number of layers; Table S5 Detailed calculations of the Griffith work for the “before diffusion” state; Table S6: Detailed calculations of the Griffith work for the “after diffusion” state. Supplementary material includes information on two different Mg/Ti interface models, i.e., Wu’s rotation model and the current vacancy model, and the surface convergence tests for (0001)_Mg_ and (0001)_Ti_. Reference [68] is cited in the supplementary materials.
