# Supplementary material for: Effect of Inherent Mg/Ti Interface Structure on Element Segregation and Bonding Behavior: An Ab Initio Study
Source: Materials (Basel). 2025 Jan 16;18(2):409. doi: 10.3390/ma18020409 (PMC11767061; doi:10.3390/ma18020409)
Supplement: Supplementary file 1 [file materials-18-00409-s001.zip › materials-3362582-supplementary.pdf]

materials

MDPI

*Supplemental Materials*

# Effect of Inherent Mg/Ti Interface Structure on Element Segregation and Bonding Behavior: An Ab Initio Study

**Xiaodong Zhu [1], Kaiming Cheng [1,*], Jin Wang [1,*], Jianbo Li [2], Jingya Wang [3], Huan Yu [1], Jixue Zhou [1,*] and Yong Du [4]**

## 1. Griffith work and interface energy for rotation model and vacancy model

We further established two Mg/Ti interface models, i.e. Wu's rotation model [1] and the current vacancy model, as shown in Fig. S1. The interfaces are represented by Mg and Ti slabs, each consisting of 6 layers stacked periodically along the c-axis with a 10 Å vacuum layer. Detailed calculations of the Griffith work and interface energy for these models are provided in Table S1 and Table S2.

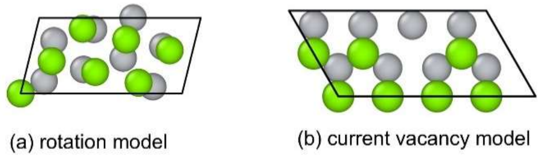


Figure. S1 Cross section of Mg-Ti interface: (a) rotation model [1], (b) vacancy model

## 2. Surface convergence

To accurately model the Mg/Ti interface, it is crucial that the slabs used in the simulation are sufficiently thick to exhibit bulk-like properties, as the adhesion characteristics of thin films can differ significantly from those of the bulk material. Therefore, we first determined the minimum thickness for a bulk-like slab by calculating surface energy's convergence with increasing slab thickness. In the supercell, the two free surfaces of the slab were separated by an 18 Å vacuum layer. The surface free energy, $\sigma_{sv}$, was calculated using the following equation [2]:

$$\sigma_{\mathrm{sv}} = \frac{E_{\mathrm{slab}}^{N} - NE_{\mathrm{bulk}}}{2S} \tag{S1}$$

where $E_{\mathrm{slab}}^{N}$ is the total energy of an N-layer slab, $E_{\mathrm{bulk}}$ is obtained by fitting a straight line to all of the $E_{\mathrm{slab}}^{N}$ data points N and to taking its slope after excluding the thinnest slabs, and S represents the surface area of the unit cell.

Table S3 and Table S4 present the calculated results of the surface energies of $(0001)_{Mg}$ and $(0001)_{Ti}$ slabs with thicknesses ranging from 3 to 9 layers.

Table S1 Calculated Griffith work for rotation model and vacancy model

| | $E_{Mg-Ti-\mathrm{interface}}\ (eV)$ | $E_{Ti-\mathrm{surface}}\ (eV)$ | $E_{Mg-\mathrm{surface}}\ (eV)$ | $A$ (Å$^2$) | $W_{\mathrm{G}}$ (J/m$^2$) |
|---|---|---|---|---|---|
| rotation model | -425.317 | -357.472 | -60.138 | 59.899 | 2.061 |
| vacancy model | -430.893 | -356.780 | -65.658 | 63.011<br>3 | 2.150 |

Table S2 Calculated interface energy for rotation model and vacancy model

| | $E_{Mg/Ti}(eV)$ | $\mu_{Mg}(eV)$ | $N_{Mg}$ | $\mu_{Ti}(eV)$ | $N_{Ti}$ | $\sigma_{Mg}$ (J/m$^2$) | $\sigma_{Ti}$ (J/m$^2$) | S (Å$^2$) | $\gamma_{int}$ (J/m$^2$) |
|---|---|---|---|---|---|---|---|---|---|
| rotation model | -425.317 | -1.544 | 42 | -7.762 | 48 | 0.559 | 1.958 | 59.899 | 0.72 |
| vacancy model | -430.893 | -1.544 | 47 | -7.762 | 48 | 0.559 | 1.958 | 63.0113 | 1.10 |

Table S3 Calculated surface energies for Mg (0001) with respect to the number of layers

| No. of layers | $E^N_{\text{slab}}(eV)$ | $E_{\text{bulk}}(eV)$ | S (Å$^2$) | $\sigma_{Mg}$ (J/m$^2$) Mg (0001) |
|---|---|---|---|---|
| 3 | -16.150 | -1.544 | 66.440 | 0.573 |
| 5 | -28.493 | -1.544 | 68.231 | 0.559 |
| 7 | -40.814 | -1.544 | 69.123 | 0.559 |
| 9 | -53.134 | -1.544 | 69.503 | 0.563 |

Table S4 Calculated surface energies for Ti (0001) with respect to the number of layers

| No. of layers | $E^N_{\text{slab}}(eV)$ | $E_{\text{bulk}}(eV)$ | S（Å$^2$） | $\sigma_{Ti}$ (J/m$^2$) Ti (0001) |
|---|---|---|---|---|
| 3 | -85.817 | -7.762 | 57.44684 | 2.045 |
| 5 | -117.007 | -7.762 | 58.6347 | 1.958 |
| 7 | -148.082 | -7.762 | 58.87098 | 1.960 |
| 9 | -179.070 | -7.762 | 58.99496 | 1.959 |

## 3. Griffith work for different doping conditions at the Mg/Ti interface

Table S5 Detailed calculations of the Griffith work for the "before diffusion" state

| X | $E_{Mg/X-Ti-\text{interface}}(eV)$ | $E_{Ti-\text{surface}}(eV)$ | $E_{Mg/X-surface}(eV$ | $A$(Å$^2$) | $W_{\text{G}}$(J/m$^2$) |
|---|---|---|---|---|---|
| Mg | -537.006 | -442.333 | -81.797 | 94.390 | 2.186 |
| Y | -541.702 | -442.094 | -86.721 | 94.927 | 2.175 |
| Gd | -539.852 | -442.084 | -84.863 | 94.941 | 2.178 |

Table S6 Detailed calculations of the Griffith work for the "after diffusion" state

| X | $E_{Mg/X-Ti-\text{interface}}(eV)$ | $E_{Ti-\text{surface}}(eV)$ | $E_{Mg/X-surface}(eV$ | $A$(Å$^2$) | $W_{\text{G}}$(J/m$^2$) |
|---|---|---|---|---|---|
| Mg | -537.334 | -442.303 | -81.790 | 94.441 | 2.246 |
| Y | -542.014 | -442.074 | -85.974 | 94.808 | 2.360 |

| | | | | | |
|---|---|---|---|---|---|
| Gd | -540.140 | -442.050 | -84.082 | 94.843 | 2.366 |

**References**

[1] Wu Y, Li X, Chen Z, Yu J, Zhao YJ (2018) Mg-X (X= Ni, Pd, Ti, Nb) interface and atomic mixture effect: A first-principles study. Mater. Res. Express 6:016305. doi:10.1088/2053-1591/aae562

[2] Fiorentini V, Methfessel MJ (1996) Extracting convergent surface energies from slab calculations. J. Phys.: Condens.Matter 8:6525-6529. doi:10.1088/0953-8984/8/36/005
